# Dietary supplementation with xylooligosaccharides and exogenous enzyme improves milk production, energy utilization efficiency and reduces enteric methane emissions of Jersey cows

**DOI:** 10.1186/s40104-023-00873-w

**Published:** 2023-06-12

**Authors:** Lifeng Dong, Lei Zhao, Bowei Li, Yanhua Gao, Tianhai Yan, Peter Lund, Zhuofan Liu, Qiyu Diao

**Affiliations:** 1 Institute of Feed Research/Sino-US Joint Lab On Nutrition and Metabolism of Ruminant, Chinese Academy of Agricultural Sciences, Beijing, 100081 China; 2 School of Agriculture, Ningxia University, Yinchuan, 750000 China; 3 College of Life Science and Technology, Southwest Minzu University, Chengdu, 610041 China; 4 Agri-Food and Biosciences Institute, Hillsborough, Co. Down BT 26 6DR UK; 5 Department of Animal Science, Aarhus University, AU Foulum, PO Box 50, 8830 Tjele, Denmark

**Keywords:** Energy utilization efficiency, Enteric methane emissions, Exogenous enzyme, Jersey cows, Xylooligosaccharides

## Abstract

**Background:**

Sustainable strategies for enteric methane (CH_4_) mitigation of dairy cows have been extensively explored to improve production performance and alleviate environmental pressure. The present study aimed to investigate the effects of dietary xylooligosaccharides (XOS) and exogenous enzyme (EXE) supplementation on milk production, nutrient digestibility, enteric CH_4_ emissions, energy utilization efficiency of lactating Jersey dairy cows. Forty-eight lactating cows were randomly assigned to one of 4 treatments: (1) control diet (CON), (2) CON with 25 g/d XOS (XOS), (3) CON with 15 g/d EXE (EXE), and (4) CON with 25 g/d XOS and 15 g/d EXE (XOS + EXE). The 60-d experimental period consisted of a 14-d adaptation period and a 46-d sampling period. The enteric CO_2_ and CH_4_ emissions and O_2_ consumption were measured using two GreenFeed units, which were further used to determine the energy utilization efficiency of cows.

**Results:**

Compared with CON, cows fed XOS, EXE or XOS + EXE significantly (*P* < 0.05) increased milk yield, true protein and fat concentration, and energy-corrected milk yield (ECM)/DM intake, which could be reflected by the significant improvement (*P* < 0.05) of dietary NDF and ADF digestibility. The results showed that dietary supplementation of XOS, EXE or XOS + EXE significantly (*P* < 0.05) reduced CH_4_ emission, CH_4_/milk yield, and CH_4_/ECM. Furthermore, cows fed XOS demonstrated highest (*P* < 0.05) metabolizable energy intake, milk energy output but lowest (*P* < 0.05) of CH_4_ energy output and CH_4_ energy output as a proportion of gross energy intake compared with the remaining treatments.

**Conclusions:**

Dietary supplementary of XOS, EXE or combination of XOS and EXE contributed to the improvement of lactation performance, nutrient digestibility, and energy utilization efficiency, as well as reduction of enteric CH_4_ emissions of lactating Jersey cows. This promising mitigation method may need further research to validate its long-term effect and mode of action for dairy cows.

**Supplementary Information:**

The online version contains supplementary material available at 10.1186/s40104-023-00873-w.

## Introduction

Mitigation of enteric methane (CH_4_) emissions is getting increasing attention as it is of great importance for alleviation of anthropogenic greenhouse gases (GHG) emissions and improvement of energy utilization efficiency of ruminant production. A recent report showed that ruminant livestock production was responsible for 56% of total agricultural GHG emissions and 93% of all livestock GHG emissions globally [1]. In addition, formation of enteric CH_4_ in the rumen of dairy cows represents an energetic loss ranging from 2.7% to 9.8% gross energy (GE) intake [2]. Nowadays, there is an increasing pressure to reduce CH_4_ emissions from all sectors of the economy as the recent Global Methane Pledge initiated at the 26^th^ session of the Conference of the Parties (COP26) set tasks to curb CH_4_ emissions by 30% below 2020 levels by 2030. Thus, due to substantial volumes of enteric CH_4_ emission, the implementation of this pledge will have major consequences for the sustainability of ruminant production regimes.

Various sustainable CH_4_ mitigation strategies have been investigated with considerations of their impact on feed conversion efficiency, animal performance, and economic feasibility. Feeding dairy cows diets supplemented with exogenous enzymes (EXE) for improvement of production efficiency have been extensively examined, whereas the role of EXE on reduction of enteric CH_4_ emissions is not fully verified. For example, Arriola et al. [3] found that dietary supplementation of fibrolytic enzyme significantly reduced CH_4_ emission of dairy cows, irrespective of whether enzymes were supplemented in low (33%) or high (48%) amounts to the concentrate diet. McGinn et al. [4] reported that the supplementing enzymes into the diet of growing beef cattle reduced enteric CH_4_ emission, CH_4_ emission as a proportion of dry matter (DM) intake, or GE intake. In an in vitro experiment, Vallejo-Hernández et al. [5] found that CH_4_ emission (mL/g DM) decreased by 24.0% as xylanase was supplemented into the anaerobic incubation system for 48 h. However, some inconsistent results were observed in that exogenous fibrolytic enzymes were considered to be degraded by the ruminal microbial communities. Zhou et al. [6] showed that CH_4_ emission remained similar when exogenous fibrolytic enzymes (endoglucanase and xylanase) were added into the diet, and the quantitative real-time PCR results indicated that the overall density and activity of ruminal methanogens were similar to the control group. In addition, the differences in composition and activities of enzymes would contribute to the inconsistency results of CH_4_ emissions of dairy cows [7, 8].

Prebiotics has lately received tremendous attention because it can be selectively used by host microorganisms providing health and production benefits [9]. The capability of prebiotics to improve fiber digestion and production performance has been reported in recent years [10, 11]. Xylooligosaccharides (XOS) is an emerging prebiotics that preferentially enhance the growth or activity of advantageous bacteria (e.g., *Bifidobacterium*) in the gastrointestinal tract. The inclusion of XOS in the diet showed positive influence on nutrient digestibility, immune function, and growth performance of monogastric animals [12, 13]. Cangiano et al. [11] found that supplementation of oligosaccharides had positive effects on growth, feed efficiency and health condition of young ruminants, which could be attributed to the enhancement of gut microbial composition and digestive efficiency [10]. Herrick et al. [14] examined the digestive and production characteristics of lactating dairy cows when hemicellulose extract (consisting of XOS as the major components) was supplemented. The results showed that this product significantly increased neutral detergent fiber (NDF) digestibility and energy utilization efficiency and would be a promising way to reduce CH_4_ emission. Similar to XOS, supplementation of galacto-oligosaccharides significantly improved growth performance and nitrogen utilization of dairy cows, and reduced enteric CH_4_ emission and CH_4_ energy output [15]. However, limited information is available regarding the effect of XOS, EXE and their combination on production performance as well as enteric CH_4_ emissions of dairy cows. Therefore, we hypothesized that supplementing with either XOS, EXE, or their combination may improve milk production and composition, nutrient digestibility, energy utilization efficiency, and reduce CH_4_ emission and intensities.

## Materials and methods

### Animals, experiment design, and management

This study was conducted between July and August 2021, at the NewHope dairy farm in Qingbaijiang district of Chengdu, China (104°22′N, 30°89′W). Ambient temperature (min. = 20.0 °C, max. = 31.5 °C, average = 24.3 °C) and relative humidity (min. = 20%, max. = 46%, average = 27%) were recorded hourly throughout the experiment (Fluke 971 Temperature Humidity Meter, Fluke Corporation, Shanghai, China). Forty-eight multiparous Jersey dairy cows with days in milk (DIM) of 160 ± 10.6 d (mean ± SD), milk yield of 22.2 ± 1.58 kg (mean ± SD), and average body weight (BW) of 464 ± 34.8 kg (mean ± SD) at the start of the experiment were used. The cows were blocked into 12 block of 4 animals/block based on BW, milk yield, and DIM, and the 4 animals within each block were then randomly assigned to 1 of 4 treatments: (1) control (basal TMR diet, CON), (2) CON with 25 g/d XOS (XOS), (3) CON with 15 g/d EXE (EXE), and (4) CON with 25 g/d XOS and 15 g/d EXE (XOS + EXE). The XOS used in the present study was provided by Yicong Bio-Tech Co., Ltd. (Zhengzhou, Henan, China) that was generated using steam-exploded corncobs. This XOS product contained 35% XOS with 65% maltodextrin as carrier [16]. The EXE was purchased from Sunson Enzyme Co., Ltd. (Yinchuan, Ningxia, China), containing cellulase (EC 3.2.1.8; 2.5 × 10^6^ units/kg), xylanase (EC 3.2.1.8; 0.2 × 10^8^ units/kg), β-glucanase (EC 3.2.1.151; 1.25 × 10^7^ units/kg), and β-mannanase (EC 3.2.1.78; 0.2 × 10^7^ units/kg) from *Trichoderma reesei*, *Trichoderma reesei*, *Bacillus subtilis*, and *Aspergillus niger*, respectively. The recommended dose of compound enzyme for lactating cows is 15 g/d per cow based on the previous results in our research team.

The experimental period lasted 60 d, with the first 14 d (from d 1 to 14) for adaptation and 46 d (from d 15 to 60) for sampling and data collection. The dietary composition and nutrient contents of the feed ingredients are presented in Table 1. All ingredients were mixed in a TMR and offered to cow twice daily: between 0600 and 0800 h, and between 1600 and 1800 h. The XOS and EXE were fed using a top-dressed method into TMR twice a day and confirmation of fully consumption of these additives was by visual observation [17]. Before the beginning of this experiment, an area from the farm was set up to keep cows in individual unit with concrete floors and clean rice husk bedding, so as to provide the TMR and collect the refusals from each cow. The barn has good ventilation and the cows had free access to water throughout the entire experiment.

**Table 1:** Ingredients and chemical composition fed to lactating Jersey cows during the study

Item	Diet
Ingredient, g/kg DM	
Corn silage	423
Alfalfa hay	124
Oat hay	50
Whole cottonseed	26
Beet pulp	39
Fat powder^1^	7
Sodium bicarbonate	3
Molasses	26
Steam-flaked corn	107
Concentrate, commercial^2^	180
Vitamin and mineral mix^3^	15
Chemical composition	
DM, g/kg (as fed)	618
OM, g/kg DM	920
Crude ash, g/kg DM	79.6
CP, g/kg DM	166
RDP, g/kg DM^4^	91.4
RUP, g/kg DM^5^	74.6
NDF, g/kg DM	323
ADF, g/kg DM	212
Ether extract, g/kg DM	40.1
Calcium, g/kg DM	7.3
Phosphorus, g/kg DM	3.4
Starch, g/kg DM	264
Utilizable CP, g/kg DM^6^	100.7
NFC, g/kg DM^7^	391
NE_L_, Mcal/kg DM^8^	173

^1^Fat powder of palm oil Polyfat K200 (Britz (Xiamen) Trading Co., Ltd., Fujian province, China)

^2^Concentrate Dairy Jersey III (Guanghan Guoxiong Foodstuff Co., Ltd., Sichuan province, China): 27% corn, 32.4% soybean meal, 8% cottonseed meal, 15% rapeseed meal, 0.83% sodium bicarbonate, 5.5% corn gluten meal, 10.5% bypass soybean meal, 0.18% sodium chloride, 0.6% magnesium oxide

^3^Mineral mix contained 26.4% CP, 5.06% Ca, 10.7% Na, 6.8% K, 4.1% Mg, 0.26% S, 1.6% P, 417 mg/kg of Mn, 665 mg/kg of Zn, 229 mg/kg of Cu, 2,166 mg/kg of Fe, 24 mg/kg of Co, 14 mg/kg of I, 7.1 mg/kg of Se, 116,511 IU of vitamin A/kg, 13,100 IU of vitamin D_3_, and 1,164 IU of vitamin E/kg (DM basis)

^4,5^RDP (Rumen-degradable protein, g/kg DM) and RUP (Rumen-undegradable protein, g/kg DM) is calculated based on tabular value (NRC, 2001) [18]

^6^Utilizable CP (uCP, g/kg DM) is calculated using RUP (g/kg DM), CP (g/kg DM) and ME (Metabolizable energy, MJ/kg DM) content as described by Edmunds et al. [19]

^7^NFC (Non-fiber carbohydrates, g/kg DM) is calculated as follows: 100 − [CP + Ash + ether extract + NDF]

^8^NE_L_ (Net energy for lactation, Mcal/kg DM) is calculated using values from Feeding Standard of Dairy Cattle [20]

### Data and sample collection

Total feed intake of each cow was recorded as the difference between feed offered and the orts throughout the experiment. The concentrate samples were collected twice per week and pooled within week, and samples of the fresh forages, TMR, and orts were collected daily and stored at −20 °C for further chemical composition analysis.

Cows were milked twice daily at 0600 and 1600 h, and milk yield was electronically recorded throughout the experiment with a DeLaval milk meter (MM25, DeLaval International, Tumba, Sweden) in a herringbone milk parlor. Milk samples were collected on d 4, 5, 6 and 7 of each week during the experimental period from morning and afternoon milking. Milk samples in the last week were collected from d 58 to 60 due to the termination of the experiment. The milk yield and composition were averaged by week for statistical analyses. Milk samples were preserved with 2-bromo-2-nitropropane-1,3 diol in special plastic tubes for analysis of milk protein, fat, lactose, and milk urea nitrogen (MUN) concentration using a MilkoScan FT6000 Milk Analyzer (Foss Inc., Hillerød, Denmark). Composited milk samples were frozen (−20 °C) until analysis for GE and N as described by Morris and Kononoff [21]. Fecal samples (approximately 100 g) were collected from the rectum of each cow at various time points (from d 20 to 24) to obtain representative samples (d 20: 1000, 1800, and 2200 h; d 21: 0200, 1000, and 1400 h; d 22: 0500,1300, and 1700 h; d 23: 0800, 1600, and 2000 h; d 24: 1100, 1900, and 2300 h). Fecal samples were pooled within cow and frozen at −20 °C for further chemical composition analysis.

Enteric CH_4_ and CO_2_ emissions and O_2_ consumption were measured using two sets of the GreenFeed Large Animal System (product No. 157 and 158; C-Lock, Inc., Rapid City, SD, USA) as described by Jia et al. [22]. Generally, 10 Jersey cows from each treatment (40 cows in total) were randomly selected and trained for 10 d to adapt the GreenFeed systems before the commencement of the experiment. To attract the cows to the GreenFeed systems, eight drops of 30 g concentrate pellet every 40 s were provided during each visit. The intake of concentrate from the GreenFeed systems was taken into account when the total DM intake and energy intake were calculated during the experiment. Gas sensor calibration of the GreenFeed systems was performed once a week using a zero gas, 100% N_2_ (99.999% pure) and span gas (a mixture of CO_2_, CH_4_, and O_2_). A CO_2_ recovery test was conducted every other week during the whole measurement; the mean (± SE) recovery was 101% ± 1.6%. Cows were allowed to access the GreenFeed systems via a unique radio-frequency identification ear tag. The gas concentration (O_2_, CH_4_ and CO_2_) were automatically measured using a paramagnetic O_2_ analyzer and nondispersive infrared CH_4_ and CO_2_ sensors. The systems were programmed to allow each cow to visit at minimum 3-h intervals. During each visit, the head position of cows remained relatively stable for more than 3 min (averaging 7 min 56 s for the present study) as a valid visit, while inappropriate gas samples (less than 3 min) were filtered out by the system. Gases were measured for 14 d (from d 32 to 44) to ensure 20 valid measurements for each cow for production of repeatable and reliable averaged daily CH_4_ emissions.

### Chemical analysis and calculations

The DM contents of feed and feces were determined by oven drying at 65 °C for 48 h. The samples were then milled using a Cyclotec mill (Tecator 1093, Tecator, Hogannas, Sweden) to pass through a 1-mm sieve for further chemical analysis. The NDF and acid detergent fiber (ADF) content were determined using heat-stable α-amylase and sodium sulfite in Ankom200 Fiber Analyzer (Ankom Technology Corp., Fairport, NY, USA) [23]. Crude protein (CP) concentration was calculated as total N concentration × 6.25. Ether extract and ash content was determined according to AOAC International [24] method 920.39 and 942.05. Gross energy (GE) content was determined using bomb calorimetry (1108 Oxygen bomb, Parr Instruments, Moline, IL, USA). The concentration of AIA in feed and feces was analyzed in both feeds and feces as an internal digestibility marker for calculation of nutrient digestibility [25]. Apparent total-tract DM digestibility was calculated from estimated intake and excretion of DM from feed and feces as described by Karlsson et al. [26] according to Eq. 1:

1 $$DM\;digestibility\;(\%)=1-(A/B)\times100,$$

where A and B were the AIA concentrations in the feed and feces, respectively. The nutrient digestibility was calculated according to Eq. 2:

2 $$Nutrient\;digestibility\;(\%)=1-\lbrack(A/B)\times(BY/AX)\rbrack\times100,$$

where AX and BY were the nutrient concentrations in the feed and feces, respectively. The energy-corrected milk yield (ECM) was calculated as described by Sjaunja et al. [27] according to Eq. 3:

3 $$ECM\;(kg/d)=milk\;yield\;(kg/d)\times\lbrack38.3\times fat\;(g/kg)+24.2\times true\;protein\;(g/kg)+16.54\times lactose\;(g/kg)+20.7\rbrack/3,140,$$

where fat, true protein, and lactose are the concentrations of these constituents in milk. Feed efficiency was calculated as milk yield (kg/d)/DM intake (kg/d) or ECM (kg/d)/DM intake (kg/d). The utilizable crude protein (uCP) content of TMR was calculated as described by Edmunds et al. [19] according to Eq. 4:

4 $$uCP\;(g/kg\;DM)=\lbrack11.93-6.82\times(RUP/CP)\rbrack\times ME+(1.03\times RUP),$$

where RUP (ruminal undegraded feed CP) and CP are in g/kg DM and ME (metabolizable energy) is in MJ/kg DM. RUP (% CP) is calculated based on tabular value (NRC, 2001) [18]. Metabolizable energy intake (MJ/d) was calculated as: ME intake (MJ/d) = GE intake (MJ/d) – fecal energy (MJ/d) – CH_4_-E (MJ/d) – urinary energy (MJ/d). Fecal energy (FE) output was calculated by using GE intake and estimated GE digestibility as described by Ramin and Huhtanen [28], where the GE digestibility was calculated using OM digestibility as described by Fant et al. [29] according to Eq. 5:

5 $$FE\;(MJ/d)=GE\;intake\;(MJ/d)-GE\;intake\times(GE\;digestibility/1,000),$$

where GE digestibility was estimated from OM digestibility as described by Ramin and Huhtanen [28] according to Eq. 6:

6 $$GE\;digestibility\;(kJ/MJ)=977\times OM\;digestibility-11.3,$$

Methane energy output (CH_4_-E) was calculated as follows: CH_4_-E (MJ/d) = CH_4_ (g/d) × 55.65 (MJ/kg)/1,000. Urinary energy output (UE) was calculated based on DM intake, forage proportion and CP content as described by Guinguina et al. [30] according to Eq. 7:

7 $$UE\;(MJ/d)=-3.6+0.37\times DM\;intake\;(kg/d)+0.006\times forage\;proportion\;(g/kg\;of\;DM)+0.03\times CP\;(g/kg\;of\;DM),$$

The efficiency of ME use for lactation (*k*_*l*_) was calculated as described by AFRC [31] according to Eq. 8:

8 $$k_l=E_{1(0)}/(ME\;intake-ME_m),$$

where E_1(0)_ is milk energy output (E_1_) adjusted to zero energy balance (MJ/d).

### Statistical analysis

Data for feed intake and milk yield were analyzed using the PROC MIXED procedure of SAS (version 9.2.0, SAS Institute Inc.), with dietary treatment considered as a fixed effect and cows as random effect. Variables with repeated measurements over time (e.g., DM intake, milk yield, milk component content) and all other measurements were reduced to experimental means for each cow. Gas emissions values were from 10 cows out of 12 cows in each treatment. Duncan’s multiple range tests were conducted when a significant difference was detected among means. Data are presented as mean ± SEM and a *P*-value of < 0.05 is considered significant.

## Results

### Feed Intake, milk yield and composition

Feed intake, milk yield and composition, and feed efficiency of Jersey cows fed the experimental diets are presented in Table 2. The BW and DM intake for the Jersey cows was similar (*P* > 0.15) among treatments at 453.3 ± 7.194 kg and 17.6 ± 0.192 kg/d, respectively. Cows fed XOS, EXE or XOS + EXE had higher (*P* < 0.05) milk yield and ECM than CON. Similar results were observed for true protein, fat and lactose content (*P* < 0.05), whereas cows fed EXE tended to increase (*P* = 0.09) MUN concentration compared with cows fed the remaining treatments. The EXE supplement demonstrated greater (*P* < 0.01) ECM/DM intake compared with cows fed the remaining treatments.

**Table 2:** Effects of xylooligosaccharides (XOS), exogenous enzyme (EXE) and their combinations on dry matter intake, milk production, and feed efficiency in lactating Jersey cows

Item	CON^1^	XOS	EXE	XOS + EXE	*P*-value
BW, kg	458 ± 79.1	446 ± 62.0	463 ± 86.1	447 ± 59.7	0.16
DM intake, kg/d	17.5 ± 1.71	17.9 ± 1.59	17.7 ± 1.82	17.4 ± 2.37	0.06
Milk yield, kg/d	18.4 ± 0.41^b^	19.6 ± 1.06^a^	18.9 ± 0.75^a^	19.4 ± 1.10^a^	< 0.01
ECM^2^, kg/d	22.4 ± 1.52^c^	24.6 ± 1.38^a^	23.4 ± 0.66^b^	23.2 ± 1.42^b^	< 0.01
True protein, %	3.87 ± 0.106^b^	4.14 ± 0.227^a^	4.07 ± 0.219^a^	4.05 ± 0.502^a^	0.04
Fat, %	5.43 ± 1.142^b^	5.56 ± 2.073^a^	5.57 ± 1.471^a^	5.52 ± 1.511^a^	0.02
Lactose, %	3.9 ± 0.48^b^	4.1 ± 0.95^a^	4.2 ± 0.65^a^	4.0 ± 0.44^a^	0.04
MUN, mg/dL	15.8 ± 1.34	16.2 ± 2.15	15.0 ± 1.37	16.0 ± 2.11	0.09
Milk yield/DM intake	1.08 ± 0.099^b^	1.12 ± 0.251^a^	1.07 ± 0.228^b^	1.11 ± 0.248^a^	0.040
ECM/DM intake	1.28 ± 0.164^c^	1.37 ± 0.173^a^	1.31 ± 0.306^b^	1.32 ± 0.131^b^	< 0.01

^1^*CON* Control (no feed additives), *XOS* 25 g/d per cow, *EXE* 15 g/d per cow, *XOS* + *EXE* Combination of XOS and EXE. Data are given as mean ± standard error of means

^2^*ECM* Energy corrected milk yield, calculated according to Sjaunja et al. [27]

^a–c^Means within a row with different superscripts are significantly different (*P* < 0.05)

## Nutrient digestibility

Dietary nutrient digestibility of lactating Jersey cows fed the experimental diets are presented in Table 3. No significant difference (*P* = 0.44) was observed for DM and CP digestibility among the 4 treatments. The CP digestibility was relatively higher for cows fed XOS, EXE or XOS + EXE compared with those fed control (*P* = 0.05). However, for fiber content digestibility, highest NDF digestibility value was observed for cows fed XOS compared with cows fed the remaining treatments (*P* < 0.05), whereas supplementation of XOS, EXE, or XOS + EXE had little influence on ADF digestibility.

**Table 3:** Effects of xylooligosaccharides (XOS), exogenous enzyme (EXE) and their combinations on apparent digestibility of nutrients in lactating Jersey cows

Item	CON^1^	XOS	EXE	XOS + EXE	*P*-value
DM, %	73.8 ± 1.35	74.0 ± 1.15	74.6 ± 1.12	73.1 ± 1.42	0.44
CP, %	72.2 ± 1.71	73.3 ± 1.41	73.8 ± 1.06	72.5 ± 1.05	0.05
NDF, %	58.6 ± 1.17^c^	64.5 ± 1.94^a^	62.9 ± 1.35^b^	62.8 ± 1.26^b^	0.04
ADF, %	57.7 ± 1.44^b^	63.2 ± 1.06^a^	64.5 ± 1.51^a^	65.8 ± 1.17^a^	0.02

^1^*CON* Control (no feed additives), *XOS* 25 g/d per cow, *EXE* 15 g/d per cow, *XOS* + *EXE* Combination of XOS and EXE. Data are given as mean ± standard error of means

^a–c^Means within a row with different superscripts are significantly different (*P* < 0.05)

## Gas emissions

Gas emissions, CH_4_ yield, and CH_4_ intensities of lactating Jersey cows fed the experimental diets are presented in Table 4. Cows fed XOS, EXE or XOS + EXE had lower CH_4_ emissions compared with those fed control, whereas lowest CH_4_ emission value (364.1 g/d) was observed for cows fed XOS. No significant difference was observed for CO_2_ production and O_2_ consumption for cows fed EXE or XOS + EXE, whereas cows fed XOS demonstrated greater (*P* < 0.05) CO_2_ production and O_2_ consumption compared with cows fed the control. Cows fed XOS, EXE or XOS + EXE had lower CH_4_/DM intake, CH_4_/milk yield, and CH_4_/ECM values compared with those fed control (*P* < 0.05), and cows fed XOS demonstrated lowest CH_4_/milk yield and CH_4_/ECM compared with cows fed EXE or XOS + EXE (*P* < 0.05).

**Table 4:** Effects of xylooligosaccharides (XOS), exogenous enzyme (EXE) and their combinations on gas production and methane emission intensities in lactating Jersey cows

Item	CON^1^	XOS	EXE	XOS + EXE	*P*-value
CH_4_, g/d	399.8 ± 22.79^a^	364.1 ± 31.42^b^	374.9 ± 26.81^b^	371.3 ± 32.20^b^	0.03
CO_2_ production, g/d	7,624 ± 1,665.2^c^	9,055 ± 1,797.6^b^	10,104 ± 1,982.5^a^	11,042 ± 2,009.2^a^	0.03
O_2_ consumption, g/d	7,242 ± 1,558.0^c^	8,905 ± 1,667.8^b^	9,330 ± 1,792.9^a^	10,884 ± 1,299.4^a^	< 0.01
CH_4_/DM intake, g/kg	22.8 ± 1.96^a^	20.3 ± 2.21^b^	21.2 ± 3.57^b^	21.3 ± 2.19^b^	< 0.01
CH_4_/Milk yield, g/kg	21.7 ± 2.20^a^	18.6 ± 3.11^c^	19.9 ± 3.52^b^	19.1 ± 2.69^b^	0.03
CH_4_/ECM, g/kg	17.8 ± 1.05^a^	14.8 ± 1.77^c^	16.0 ± 2.10^b^	16.0 ± 2.51^b^	< 0.01
RQ_metab_^2^	0.58 ± 0.012	0.60 ± 0.09	0.65 ± 0.013	0.62 ± 0.014	0.07

^1^*CON* Control (no feed additives), *XOS* 25 g/d per cow, *EXO* 15 g/d per cow, and *XOS* + *EXE* Combination of XOS and EXE. Data are given as mean ± standard error of means

^2^RQ_metab_ = metabolic respiratory quotient, calculated as metabolic CO_2_ production/O_2_ consumption on a volume basis according to Derno et al. [32]

^a–c ^Means within a row with different superscripts are significantly different (*P* < 0.05)

## Energy utilization efficiency

Energy utilization of lactating Jersey cows fed the experimental diets are presented in Table 5. Dietary GE content, GE intake, and urinary energy output were not affected by treatment averaging 21.6 ± 0.38 MJ/kg, 399.9 ± 3.99 MJ/d, and 11.4 ± 0.12 MJ/d, respectively (Table 4). Significant difference was observed for fecal energy output and CH_4_-E output for cows fed XOS compared with those fed the remaining treatments (*P* < 0.05). Cows fed XOS demonstrated higher (*P* < 0.01) DE intake and ME intake compared with those fed the remaining treatments, and cows fed the EXE or XOS + EXE were intermediate and similar (*P* > 0.05). Cows fed XOS had greater (*P* < 0.05) milk energy output compared with cows fed the remaining treatments, whereas cows fed EXE or XOS + EXE demonstrated higher milk energy output (*P* = 0.03) compared with cows fed the control. Cows fed XOS had significantly higher DE intake/GE intake and milk energy output/GE intake but lower CH_4_-E output/GE intake compare with those fed the remaining treatments (*P* < 0.03). However, these energy utilization efficiency parameters (e.g., DE intake/GE intake, milk energy output/GE intake) were similar for cows fed EXE or XOS + EXE. No significant difference was also observed for* k*_*l*_ for cows fed any supplantation compared with those fed CON.

**Table 5:** Effects of xylooligosaccharides (XOS), exogenous enzyme (EXE) and their combinations on energy intake and output, and utilization efficiency in lactating Jersey cows

Item	CON^1^	XOS	EXE	XOS + EXE	*P*-value
Energy intake and output, MJ/d					
GE intake	397.3 ± 4.25	405.3 ± 6.72	401.8 ± 8.41	395.0 ± 5.22	0.72
Fecal energy output^2^	116.5 ± 1.05^a^	112.6 ± 1.80^b^	114.9 ± 2.26^a^	115.3 ± 1.57^a^	0.03
Urinary energy output^3^	11.5 ± 1.15	11.2 ± 1.46	11.5 ± 1.01	11.4 ± 1.91	0.52
CH_4_-E output	22.4 ± 1.35^a^	20.2 ± 1.01^c^	21.0 ± 1.46^b^	20.9 ± 1.71^b^	< 0.01
DE intake^4^	280.4 ± 0.46^b^	293.7 ± 0.25^a^	287.1 ± 0.77^a^	278.6 ± 0.20^b^	< 0.01
ME intake^5^	246.9 ± 2.55^c^	262.9 ± 3.26^a^	254.4 ± 3.42^b^	250.4 ± 1.89^b^	< 0.01
Milk energy output	97.5 ± 5.77^d^	110.4 ± 9.46^a^	102.1 ± 8.52^c^	104.7 ± 7.69^b^	0.03
Energy utilization efficiency					
DE intake/GE intake, %	70.6 ± 2.59^c^	72.6 ± 3.36^a^	71.5 ± 4.03^b^	70.3 ± 2.17^b^	< 0.01
CH_4_-E output/GE intake, %	5.61 ± 0.412^a^	5.01 ± 0.571^c^	5.22 ± 0.217^b^	5.25 ± 0.639^b^	< 0.01
Milk energy output/GE intake, %	24.6 ± 1.52^c^	27.2 ± 1.73^a^	25.4 ± 1.06^b^	26.5 ± 1.17^b^	< 0.01
*k*_*l*_^6^	0.614 ± 0.1170	0.629 ± 0.1461	0.613 ± 0.1352	0.620 ± 0.1191	0.47

^1^*CON* Control (no feed additives), *XOS* 25 g/d per cow, *EXO* 15 g/d per cow, and *XOS* + *EXE* Combination of XOS and EXE. Data are given as mean ± standard error of means

^2^Fecal energy output is calculated based on GE intake and estimated GE digestibility according to Ramin and Huhtanen [28]

^3^Urinary energy output is calculated according to Guinguina et al. [30]

^4^*DE* Digestible energy. DE intake is calculated as GE intake − fecal energy output

^5^*ME* Metabolizable energy. ME intake is calculated as GE intake − fecal energy output − urinary energy output − CH_4_-E output

^6^*k*_*l*_ = efficiency of ME use for lactation

^a–c^Means within a row with different superscripts are significantly different (*P* < 0.05)

## Discussion

### Feed Intake, milk yield and composition

Dietary supplementation of XOS, EXE, or a combination of XOS and EXE did not affect BW and DM intake of lactating Jersey cows, which agreed with observations from Zilio et al. [33] and Pech-Cervantes et al. [8]. Using Jersey heifers, Gandra et al. [34] found that supplementation of 20 g/d enzyme product (Fibrozyme™, Alltech, Nicholasville, KY, USA) did not affect feed intake (*P* = 0.307). Herrick et al. [14] found that Holstein cows fed a diet containing a hemicellulose extract (1.0% of diet DM) mainly consisting of XOS had no influence on DM intake, but significantly increased milk yield. However, Romero et al. [35] found a greater DM intake of diets supplemented with xylanase and cellulase at a 75:25 (v/v) mixture of Cellulase Plus and Xylanase Plus EFE (Dyadic International), suggesting that the xylanolytic capacity played a key role in hydrolyzing fiber cell walls. A meta-analysis by Arriola et al. [36] showed that feeding exogenous fibrolytic enzymes to dairy cows had little influence on DM intake, whereas large variability existed due to differences of enzyme types as well as application rates, methods, and forms of the enzyme.

Compared with CON, XOS and EXE synergistically (*P* < 0.05) increased milk yield, fat concentration, and energy-corrected milk yield (ECM)/DM intake, and XOS appeared to be more effective than EXE at increasing ECM and true protein concentration. Klingerman et al. [37] found that dietary supplementation of amylase increased milk production of dairy cows. In agreement with our results, Mohamed et al. [38] found that supplementation of a fibrolytic enzyme increased milk production by 1.5 kg/d, which was attributed to improved ruminal particulate passage rate and fiber digestion. Gado et al. [39] supplemented an enzymes mixture to Holstein dairy cows, and found that milk protein production increased by 0.12 kg/d compared with control group. These results were similar with the present experiment in which the true protein production increased by 23.6% for cows fed XOS compared with those fed CON. Consequently, we observed a higher feed conversion efficiency (milk yield/DM intake and ECM/DM intake) when EXE or XOS was supplemented.

### Nutrient digestibility

Feeding XOS, EXE, or XOS + EXE showed no significant influence on DM and CP digestibility, which was in line with previous studies [40, 41]. Refat et al. [42] reported no effect of fibrolytic enzymes rich in xylanase and cellulase on DM and OM digestibility of Holstein cows. However, supplementing XOS to the diet of Jersey cows significantly increased CP digestibility compared with cows fed CON. This result agreed with the study by Chen et al. [43] in that feeding α-amylase increased CP digestibility but had no effect on DM intake and starch digestibility. Significant difference was observed for NDF and ADF digestibility for cows fed experimental diets, which agreed with Zilio et al. [33] that feeding fibrolytic enzyme to ruminants increased NDF digestibility without affecting DM intake. Herrick et al. [14] reported that dietary supplementation of XOS increased NDF digestibility and energy utilization of Holstein lactating cows. Zilio et al. [33] reported that synergism existed between endogenous and exogenous enzymes which would contribute to the improvement of ruminal enzymatic activity, hydrolysis capacity, and thus nutrient digestibility. The EXE product was a mixture of cellulase, xylanase, β-glucanase, and β-mannanase from various microbial strains, which exerted positive effect on ruminal fermentation and nutrient digestibility in the present experiment. Moreover, Tirado-González et al. [44] reported that multi-enzyme solution may work better than extracts of almost pure enzymes, and suggested that optimized mixture of enzymes would exhibited better performance on nutrient digestibility.

### Gas emissions

Recent studies have been conducted to examine the effect of EXE supplementation on enteric CH_4_ from dairy cows, although these results were inconsistent and equivocal using either in vivo or in vitro methods [45, 46]. Mohamed et al. [38] reported that supplementation of β-glucanase, xylanase, and cellulase had little influence on in vitro CH_4_ production. In another study, Vallejo-Hernández et al. [5] found that CH_4_ yield (mL/g DM) decreased by 24.0% as xylanase was added into an in vitro evaluation system for 48 h of incubation. In a recent study, enteric CH_4_ production and CH_4_ intensity tended to increase when lactating cows were supplemented with exogenous enzyme products and CH_4_ measured using the GreenFeed system [47]. However, in agreement with our results, Arriola et al. [3] found that supplementation of fibrolytic enzymes including cellulase, xylanase, and esterase reduced CH_4_ production by 11.4% for cows fed a high-concentrate diet. Similarly, Zilio et al. [33] reported that exogenous fibrolytic enzyme supplementation reduced CH_4_ production of lactating Holstein cows.

In the present experiment, significant difference was observed for cows fed XOS, EXE, or XOS + EXE in terms of CH_4_/DM intake, CH_4_/milk yield, and CH_4_/ECM, reflecting lower CH_4_ emissions of Jersey cows fed experimental diets compared with cows fed control. Reduction of CH_4_ might be attributed to changes of ruminal fluid concentrations of total VFA or molar proportions of individual VFA, which could be used to estimate CH_4_ production in in vitro studies [3, 33]. Previous studies suggested that exogenous enzyme might affect the bacterial community and methanogenesis in the rumen, and thereby closely related to CH_4_ emission [6, 8]. Moreover, differences in enzymatic activity, application dose, and animal breed might result in inconsistent results [42]. Previously, the function of XOS was mainly related to improved nutrient digestibility and growth performance of monogastric animals or young ruminants [11]. As the major components of hemicellulose extract by steam extraction, XOS was reported to increase NDF digestibility and energy utilization of lactating dairy cows [14]. To our knowledge, the present experiment is the first report on the effect of XOS on enteric CH_4_ emissions from lactating Jersey cows. Dietary XOS supplementation significantly reduced enteric CH_4_ production and CH_4_/ECM by 12.7% and 26.0% respectively, which would be a very promising approach to reduce carbon footprint in dairy production systems. Further studies are required to examine the long-term effect and mode of action of EXE and XOS supplementation on enteric CH_4_ emissions before application.

### Energy utilization efficiency

The present study was also conducted to evaluate the effects of XOS or EXE on energy utilization efficiency using this portable automated open-circuit gas quantification system (i.e., GreenFeed system). This system was considered to produce accurate gas production values that can be used to estimate energy metabolism of dairy cows [30, 46]. The UE output as a proportion of GE intake was approximately 2.85%, and thereby similar to those reported by Morris and Kononoff [21] (2.70%–3.05%, average = 2.83%) and Uddin et al. [47] (2.48% to 3.17%, average = 2.91%) in lactating Jersey cows. Compared with CON, cows fed XOS had 3.3% and 13.1% lower FE and CH_4_-E output, which resulted in higher DE and ME intake for XOS-fed cows than CON. Milk energy output from cows fed XOS, EXE, or combination of XOS and EXE was significantly higher than those fed CON, reflecting that greater energy utilization efficiency was consistently related to lower energy losses as feces, urine, CH_4_, and heat [48].

Methane energy output expressed per unit of GE intake (*Y*_*m*_) was widely used as a key value for the calculation of regional or national enteric CH_4_ emission inventories. Previously, we reported *Y*_*m*_ values between 5.06% and 8.17% from lactating Holstein dairy cows fed with different NDF/NFC ratios [49]. Morris and Kononoff [50] summarized 15 treatments using Jersey dairy cows, and reported *Y*_*m*_ values from 3.97% to 5.01% with an average value of 4.51%, which were close to the present experiment that *Y*_*m*_ ranged from 4.8% to 5.6% (mean value = 5.18%). Another important parameter relating to energy partitioning is the efficiency of ME use for lactation (*k*_*l*_), which ranged from 0.604 to 0.629 in the present study. The NRC (2001) [18] assumes that *k*_*l*_ is approximately 0.63, and Yan et al. [51] reported that the *k*_*l*_ was between 0.61 to 0.68 for lactating Holstein–Friesian cows. Using the lactating Jersey cows, calculated *k*_*l*_ values were reported to range from 0.564 to 0.699, indicating a large range of variation [50, 52]. Higher ME intake and milk energy output from XOS, EXE or XOS + EXE treatment observed in the present study would suggest that more energy is being partitioned toward lactation, which was reflected by the relatively higher calculated *k*_*l*_ values comparted with CON. Meanwhile, some studies showed that dietary supplemented lipogenic nutrients or abomasal infusion of glucogenic substrates would cause a shift in energy partitioning toward milk production, as well as less energy requirement for maintenance [29, 53, 54]. More research may be needed to elucidate the energetic expenditure and utilization efficiency when cows are under different feeding regimes.

## Conclusions

In conclusion, this study demonstrated that dietary supplementation with XOS at 25 g/d and EXE at 15 g/d enhanced the lactation performance as well as reduced enteric CH_4_ emissions of Jersey cows. This might be because the supplementation of XOS, EXE, or combination of XOS and EXE increased nutrient digestibility and energy utilization efficiency, which was the consequence of changes of ruminal fermentation condition and composition of methanogens. Future research is warranted to evaluate the long-term effect and mode of action of these supplementations for dairy cows.

## Supplementary Information

**Additional file 1: Table S1.** The characteristic of lactating Jersey cows before entering the experiment. **Table S2.** Milk sampling during the whole experiment.

## Data Availability

Data available by the Author by request (donglifeng@caas.cn).

## Abbreviations

ADF: Acid detergent fiber
AIA: Acid insoluble ash
BW: Body weight
CH_4_: Methane
CH_4_-E: Methane energy
DIM: Days in milk
DM: Dry matter
EB: Energy balance
ECM: Energy corrected milk yield
E_l_: Milk energy output
EXE: Exogenous enzymes
FE: Fecal energy
GE: Gross energy
GHG: Greenhouse gases
*k*_*l*_: Metabolizable energy use for lactation
ME: Metabolizable energy
NDF: Neutral detergent fiber
UE: Urinary energy; HP: Heat production
UN: Nitrogen excretion
XOS: Xylooligosaccharides
*Y*_*m*_: Methane conversion factor
