# Supplementary material for: Dietary supplementation with xylooligosaccharides and exogenous enzyme improves milk production, energy utilization efficiency and reduces enteric methane emissions of Jersey cows
Source: J Anim Sci Biotechnol. 2023 Jun 12;14:71. doi: 10.1186/s40104-023-00873-w (PMC10258951; doi:10.1186/s40104-023-00873-w)
Supplement: Supplementary file 1 — Additional file 1: Table S1. The characteristic of lactating Jersey cows before entering the experiment. Table S2. Milk sampling during the whole experiment. [file 40104_2023_873_MOESM1_ESM.docx]

**Table S1** The characteristic of lactating Jersey cows before entering the experiment

| **No.** | **Groups** | **Animal No.** | **parity** | **Age, month** | **Body weight, kg** | **Days in milk, d** | **Milk yield, kg/d** |
| --- | --- | --- | --- | --- | --- | --- | --- |
| 1 | CON | 772147 | 3 | 46.9 | 452 | 229 | 21.21 |
| 2 |  | 770703 | 2 | 47.5 | 508 | 227 | 20.34 |
| 3 |  | 770678 | 2 | 47.8 | 468 | 218 | 24.58 |
| 4 |  | 770696 | 2 | 47.8 | 492 | 74 | 20.53 |
| 5 |  | 770754 | 3 | 47.1 | 515 | 74 | 24.67 |
| 6 |  | 772571 | 3 | 47.9 | 447 | 101 | 26.06 |
| 7 |  | 772734 | 3 | 47.6 | 462 | 199 | 20.86 |
| 8 |  | 770661 | 2 | 48.1 | 485 | 197 | 21.9 |
| 9 |  | 772562 | 3 | 48.0 | 473 | 196 | 21.96 |
| 10 |  | 770651 | 3 | 48.5 | 519 | 126 | 21.47 |
| 11 |  | 772570 | 2 | 47.9 | 413 | 121 | 21.75 |
| 12 |  | 772559 | 2 | 47.8 | 440 | 120 | 23.09 |
| 13 | XOS | 772703 | 2 | 47.5 | 492 | 217 | 20.47 |
| 14 |  | 772578 | 2 | 48.1 | 447 | 212 | 20.12 |
| 15 |  | 770717 | 3 | 47.6 | 484 | 211 | 22.63 |
| 16 |  | 770667 | 2 | 48.0 | 462 | 92 | 24.85 |
| 17 |  | 772750 | 3 | 47.6 | 472 | 73 | 22.82 |
| 18 |  | 772148 | 2 | 47.7 | 411 | 91 | 20.52 |
| 19 |  | 771892 | 2 | 47.7 | 482 | 192 | 21.5 |
| 20 |  | 772138 | 3 | 48.5 | 482 | 189 | 20.17 |
| 21 |  | 770686 | 2 | 48.0 | 445 | 182 | 22.59 |
| 22 |  | 770721 | 3 | 47.6 | 406 | 141 | 21.39 |
| 23 |  | 772754 | 2 | 47.5 | 502 | 146 | 23.61 |
| 24 |  | 770747 | 3 | 47.8 | 425 | 133 | 22.46 |
| 25 | EXE | 772767 | 2 | 47.2 | 501 | 211 | 24.39 |
| 26 |  | 772771 | 2 | 47.8 | 417 | 211 | 22.65 |
| 27 |  | 770742 | 2 | 47.8 | 447 | 209 | 21.78 |
| 28 |  | 772769 | 2 | 47.9 | 436 | 104 | 23.53 |
| 29 |  | 772697 | 3 | 46.5 | 505 | 94 | 21.52 |
| 30 |  | 771849 | 2 | 47.6 | 495 | 93 | 21.88 |
| 31 |  | 770720 | 3 | 46.9 | 507 | 180 | 20.76 |
| 32 |  | 772557 | 2 | 47.3 | 428 | 173 | 20.04 |
| 33 |  | 772698 | 2 | 48.1 | 465 | 170 | 20.71 |
| 34 |  | 772699 | 3 | 47.6 | 452 | 152 | 22.98 |
| 35 |  | 772140 | 2 | 48.1 | 471 | 139 | 21.29 |
| 36 |  | 770705 | 2 | 48.1 | 465 | 145 | 22.4 |
| 37 | XOS+EXE | 772150 | 2 | 46.9 | 480 | 209 | 21.35 |
| 38 |  | 772758 | 2 | 45.5 | 486 | 205 | 21.12 |
| 39 |  | 770679 | 2 | 48.0 | 475 | 200 | 22.11 |
| 40 |  | 770669 | 3 | 48.2 | 441 | 116 | 23.99 |
| 41 |  | 770652 | 2 | 48.2 | 482 | 265 | 23.41 |
| 42 |  | 770714 | 2 | 47.9 | 496 | 91 | 24.99 |
| 43 |  | 770693 | 3 | 48.0 | 412 | 163 | 20.04 |
| 44 |  | 770715 | 2 | 48.0 | 420 | 162 | 22.88 |
| 45 |  | 770692 | 3 | 47.0 | 472 | 160 | 21.51 |
| 46 |  | 770698 | 3 | 48.0 | 450 | 158 | 23.46 |
| 47 |  | 770673 | 2 | 46.8 | 411 | 155 | 20.5 |
| 48 |  | 771893 | 2 | 47.4 | 462 | 152 | 25.62 |

Note: Forty-eight multiparous Jersey dairy cows were used in the present study and divided into four groups as CON, XOS, EXE, and XOS+EXE. The homogeneity of four groups was as follows: the average age, parity, milk yield, body weight, and days in milk for CON, XOS, EXE, and XOS+EXE of lactating Jersey cows before the commencement of the experiment were 47.7 month, 2.5, 22.4 kg/d, 472.8 kg, and 156.8 d; 47.8 month, 2.4, 21.9 kg/d, 459.2 kg, and 156.6 d; 47.6 month, 2.3, 22.0 kg/d, 465.8 kg, and 156.8 d; 47.5 month, 2.3, 22.6 kg/d, 457.3 kg, and 169.7 d, respectively. Statistical analyses performed using SPSS (version 25.0, SPSS Inc.) showed no significant difference among the four groups in terms of parity, milk yield, body weight, and days in milk of cows

**Table S2** Milk sampling during the whole experiment

| **Date** | **Sampling** | | |
| --- | --- | --- | --- |
|  | **Milk yield and component** | **Fecal composition** | **Gas measurement** |
| Adaption period, from 1^st^ July to 14^th^ July (d 1–14) | - | - | - |
| Experiment period, from 15^th^ July to 29^th^ August (d 15–60) | d 16–19 | - | - |
|  | d 23–26 | d 20–24 | - |
|  | d 30–33 | - | d 32–44 |
|  | d 37–40 | - | d 32–44 |
|  | d 44–47 | - | d 32–44 |
|  | d 51–54 | - | - |
|  | d 58–60 | - | - |
